# Impact of Seed Dressing and Soil Application of Potassium Humate on Cotton Plants Productivity and Fiber Quality

**DOI:** 10.3390/plants9111444

**Published:** 2020-10-26

**Authors:** Asmat Ullah, Muqarrab Ali, Khurram Shahzad, Fiaz Ahmad, Shahid Iqbal, Muhammad Habib Ur Rahman, Shakeel Ahmad, Muhammad Mazhar Iqbal, Subhan Danish, Shah Fahad, Jawaher Alkahtani, Mohamed Soliman Elshikh, Rahul Datta

**Affiliations:** 1 Department of Agronomy, Muhammad Nawaz Sharif University of Agriculture, Multan 60000, Pakistan; ranaasmat6@gmail.com (A.U.); shahid.iqbal@mnsuam.edu.pk (S.I.); habib.rahman@mnsuam.edu.pk (M.H.U.R.); 2 Plant Nutrition Section, Mango Research Institute, Multan 60000, Pakistan; khurram.balouch2576@gmail.com; 3 Central Cotton Research Institute, Multan 60000, Pakistan; fiazdrccri@gmail.com; 4 Institute of Crop Science and Resource Conservation (INRES), University Bonn, 53115 Bonn, Germany; 5 Department of Soil and Environmental Sciences, Muhammad Nawaz Sharif University of Agriculture, Multan 60000, Pakistan; shakeel.ahmad@mnsuam.edu.pk; 6 Soil and Water Testing Laboratory for Research Chiniot, Department of Agriculture, Faisalabad 38000, Pakistan; mazhar1621@hotmail.com; 7 Department of Soil Science, Faculty of Agricultural Sciences and Technology, Bahauddin Zakariya University, Multan 60800, Pakistan; 8 Hainan Key Laboratory for Sustainable Utilization of Tropical Bioresource, College of Tropical Crops, Hainan University, Haikou 570228, China; 9 Department of Agronomy, The University of Haripur, Haripur 22620, Pakistan; 10 Department of Botany and Microbiology, College of Science, King Saud University, Riyadh 11451, Saudi Arabia; jsalqahtani@ksu.edu.sa (J.A.); melshikh@ksu.edu.sa (M.S.E.); 11 Department of Geology and pedology, Faculty of forestry and wood technology, Mendel University in Brno, Zemedelska 1, 61300 Brno, Czech Republic

**Keywords:** biological yield, application method, grain yield, nitrogen, phosphorus, potassium

## Abstract

Humus is the stable form of added crop and animal residues. The organic matter after a long-term decomposition process converts into humic substances. The naturally occurring humus is present in less amount in soils of the arid and semi-arid regions. The addition of commercially available humic acid can, therefore, contribute to improving soil health and crop yields. The present study was conducted to evaluate the effect of potassium humate, applied through soil seed dressing, on cotton productivity and fiber quality attributes. Seed dressing with potassium humate was done at the rate of 0, 100, 150 and 200 mL kg^−1^ seed while in soil potassium humate was applied at the rate of 0, 10, 20 and 30 L ha^−1^. Results showed that the combined application of potassium humate by seed dressing and through soil application improved the soil properties, productivity and fiber quality traits of cotton. All levels of soil applied potassium humate (10, 20 and 30 L ha^−1^) performed better over seed dressing in terms of cotton productivity and fiber quality attributes. Among the soil application rates, 20 L ha^−1^ potassium humate proved better as compared to other rates (0, 10 and 30 L ha^−1^). Higher soil application of potassium humate (30 L ha^−1^) showed depressing effects on all the traits studied like the reduction of 12.4% and 6.6% in Ginning out turn and fiber length, respectively, at a seeding dressing of 200 mL kg^−1^. In conclusion, potassium humate seed dressing and soil application at the rate of 200 mL kg^−1^ and 20 L ha^−1^, respectively, is a better approach to improve cotton productivity. Soil potassium humate should not exceed a rate of 20 L ha^−1^ when the seed dressing of potassium is also practiced.

## 1. Introduction

Humus is a stable form of organic matter in the soil. The added organic matter in the form of plant residues, animal manures and raw oxidized compounds after a long-time decomposition process convert into humic substances and this process is named humification [1]. Compost is the intermediate form of decomposition which contains humic substances and partially decomposed organic matter [2]. The humic substances are present in almost all soils in small amounts, in the form of potassium humate, potassium fulvates and humins [3]. Naturally occurring humic substances from low grade lignites and leonardites are the best fertilizer ingredients, use or organic fertilizer leads to risk of xenobiotic compound [4,5,6,7]. In the present time, humic substances are also commercially available in the form of inexpensive salts for agriculture application. The commercial potassium humate is extracted from brown coal using an alkaline extraction process.

Humus contains carbohydrates, lipids, protein, phenol-aromatic, protein-derived and cyclic nitrogen compounds [3]. However, the composition of humus depends upon abiotic controls, soil biota and the types of input crop and animal residues. The addition of organic amendments, i.e., biochar, organic matter, farmyard manure, compost and especially humic substances in soil with low organic matter status could increase the physical, chemical and biological properties of the soil [8,9,10,11,12,13,14,15,16,17]. soil characteristics vary with land use [18,19,20]. Moreover, the addition of humic substances has been found to be helpful in reducing the leaching of anion-like nitrate, sulphate and phosphate due to their high absorption capacity [3]. The increased availability of essential nutrients by the application of potassium humate has increased the growth and yield of crops of many studies. The soil application of humic substances also resulted in the improvement of soil particle aggregation and available water contents. The growth and yield of crops were increased in many studies by the use of humic acid substances due to the increase in cell membrane permeability, the stimulation of seed germination and viability, respiration, photosynthesis rate and root cell elongation, increasing the activity of enzymatic antioxidants such as catalase (CAT), peroxidase (POD) and superoxide dismutase (SOD). These enzymes play an important role in activating the formation of structural protein [3,21,22,23,24]. Furthermore, humic substances can increase the biotic and abiotic stress tolerance capacity of the crop plant.

In Pakistan, cotton is the second largest crop in terms of area after wheat production. Cotton contributes 1% in terms of GDP and 6.5% in agriculture value addition [25]. Cotton is the main source of foreign exchange and it provides raw material to the textile sector. Being a tropical crop, cotton production requires an average temperature from 20 to 30 °C during its growth and development [26]. However, in Multan, Pakistan, the average temperature remains from 25 to 35 °C during cotton growing season. Higher temperature than the aforementioned range causes the decomposition of organic matter at an accelerated rate. Therefore, the decomposition of organic matter and its escape in the atmosphere in the form of CO_2_ in cotton growing areas results in the decline of organic matter status and soil fertility [27]. In Pakistan, organic matter status due to prevailing high temperatures and arid climate comes down below 1% in most soils [28,29]. Therefore, the addition of crop residues only to increase the soil fertility in the soil of high temperature has not proved to be very successful [27]. The use of commercially available stable organic compounds like potassium humate can be a viable option to increase soil organic matter content and fertility on a sustainable basis. By increasing the soil organic matter status and soil fertility, the production of cotton crop and fiber quality could be increased up to a maximum extent [30,31].

Patil et al. [32] evaluated the effect of potassium humate on the seed germination and seedling growth (root length and shoot length) of wheat in laboratory conditions. Wheat seeds were treated with potassium humate at ten different concentrations (from 0.1 to 1%) and distilled water served as the control. The results showed the positive effects of applied potassium humate as an increase in seed germination, the root and shoot length of wheat were recorded compared to the control.

In another study, Kumar et al. [33] evaluated the yield response of rice to the applied potassium humate and chemical fertilizers. Potassium humate was applied at 0,5 and 10 mg kg^−1^ soil with 100 and 75% recommended doses of nitrogen phosphorus and potassium (NPK) fertilizers (60, 30, 30 mg kg^−1^, respectively). The application of potassium humate at 10 mg kg^−1^ with NPK fertilizers resulted in a significant increase in plant height, number of tillers, panicle height, panicle length, straw yield and grain yield of rice. Basbag [34] studied the effect of humic acid treatment (foliar spray, seed soaking, seed soaking + foliar spray) on cotton productivity. The increase in cotton seed yield, sympodial branches, number of bolls, fiber length and fiber fitness were higher where the seed soaking of humic acid was done.

The combined application of seed dressing and soil application of potassium hamate has been rarely studied in cotton under the arid climatic conditions of Multan, Pakistan. Therefore, the present study aimed to evaluate the effect of applied potassium humate to soil, as the seed dressing or in combination with cotton growth, yield and fiber quality attributes. It was hypothesized potassium humate in combination may be more effective compared to the individual application of soil and seed dressing for the improvement of growth, yield and fiber quality attributes of cotton.

## 2. Results and Discussion

### 2.1. Weather Conditions

**Figure 1:**
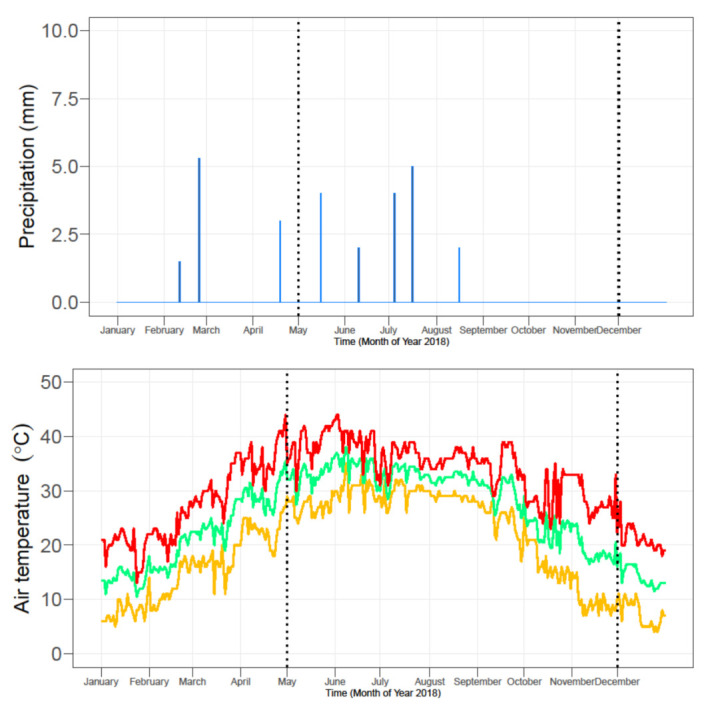
Daily total precipitation and average daily minimum (T_min_), mean (T_avg_), and maximum (T_max_) air temperature. Daily precipitation in Multan, Pakistan, from January to December 2018. The dotted lines show the duration of the experiment from the sowing to the harvesting of the cotton crop.

The weather data of rainfall and temperature were collected from the weather station located at the Central Cotton Research Institute, Multan, Pakistan, that is located 2 km away from the research site. The highest temperature of the crop season was recorded in the month of June (42 °C), while the minimum temperature was recorded in the month of November (8.1 °C). The total rainfall throughout the growing season was 106 mm (Figure 1). The irrigation water requirement of the crop was met through the canal and tube well irrigation sources.

### 2.2. Soil Properties

The application of potassium humate to soil resulted in the decrease in soil salinity (EC) while an increase in organic matter, phosphorus and potassium levels as compared to the pre-experiment status of soil (Table 1). The EC decreased by 10.0, 130.0 and 310.0 µS cm^−1^ at potassium humate levels of 10, 20 and 30 L kg^−1^ soil, respectively, over the control. The minimum value of soil pH (8.37) was recorded at a 30 L ha^−1^applied potassium humate. Applied potassium humate to soil at the rate of 30 L ha^−1^ increased organic matter by 0.50, 0.40, 0.20 g kg^−1^ soil over the potassium humate levels of 0, 10 and 20 L ha^−1^, respectively. Similarly, the available phosphorus increased by 0.19, 0.46 and 0.89 mg kg^−1^ soil while potassium increased by 12.0, 17.5 and 27.5 mg kg^−1^ soil with the application of 10, 20 and 30 L ha^−1^ potassium humate, respectively, over the control (Table 1).

### 2.3. Plant Height, Number of Nodes and Number of Sympodial Branches

**Figure 2:**
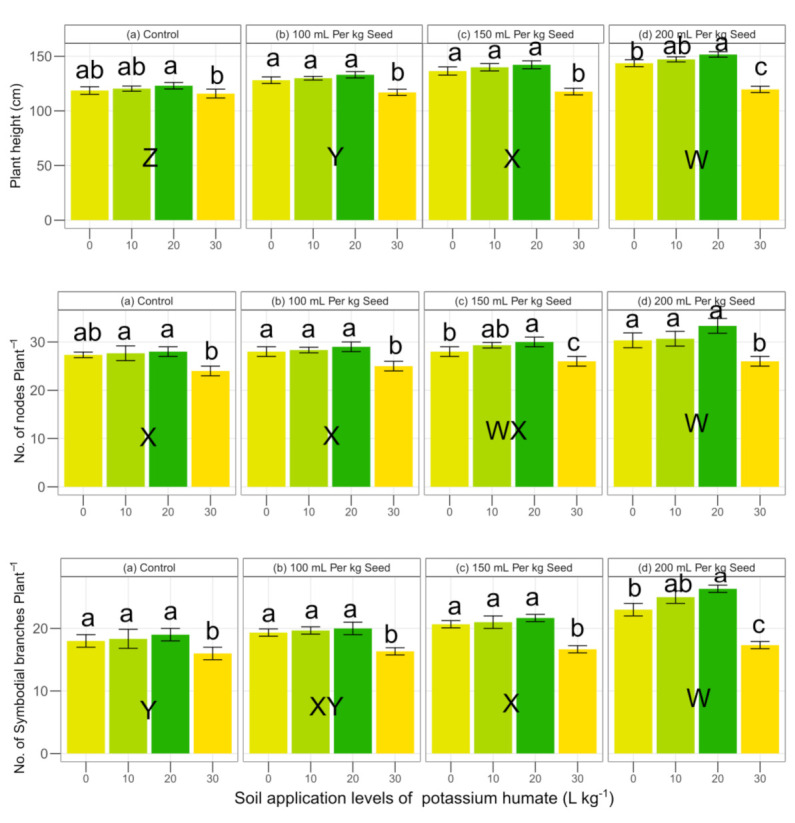
Effect of potassium humate on plant height, number of nodes and sympodial branches of cotton. Within the seed dressing application, the same lowercase letter (s) a, b, or c, indicates that the soil potassium humate application rates are not statistically different at *p* < 0.05. Among the seed dressings, the same uppercase letter (s) W, X, Y or Z, showed a non-significant effect. Error bars represent the standard deviation of the mean (n = 3, three replications).

**Figure 3:**
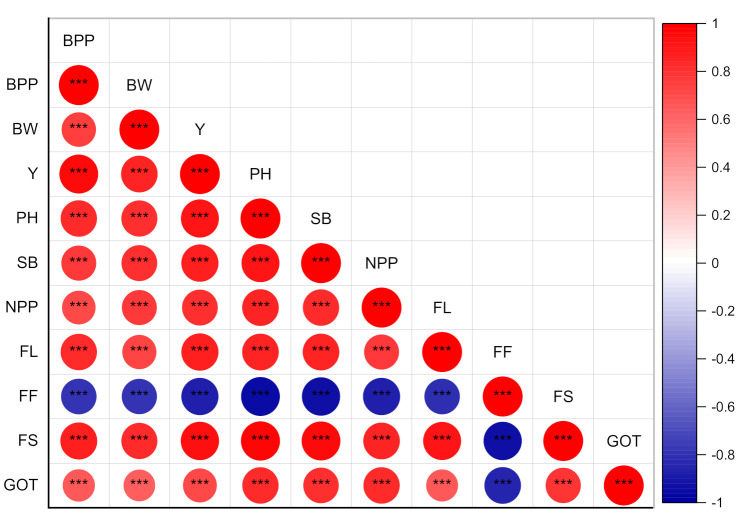
Pearson correlation of different cotton growth yield and quality attributes under different levels of soil and seed dressing potassium humate. PH = potassium humate; BPP = bolls per plant; BW = bolls weight; Y = cotton seed yield; PH = plant height; SB = sympodial branches; NPP = nodules per plant; FL = fiber length; FF = fiber fitness; FS = fiber strength; GOT = ginning out turn.

The main interactive effects of soil applied and seed dressed potassium humate were found to be significant at *p* < 0.05 on the plant height (Table 2). The soil applied potassium humate levels of 20 L ha^−1^caused a maximum plant height over other levels as well as the overall seed dressing levels of potassium humate, i.e., 0, 100, 150 and 200 mL kg^−1^ seed. However, all the levels of soil applied potassium humate (10, 20 and 30 L ha^−1^) depicted more plant height over all the doses of potassium humate applied through seed dressing (Figure 2). However, the soil potassium humate at the rate of 30 L ha^−1^ showed a lower plant height as compared to 0, 10 and 30 L ha^−1^) at all seed dressing levels (Figure 2). The main effect of potassium humate soil application and seed dressing was found to be significant at *p* < 0.05 on nodes per plant (Table 2). However, the interaction effect of soil application × seed dressing was found to be non-significant at *p* < 0.05 on nodes per plant (Table 2). The soil application of potassium humate at the rate of 20 L ha^−1^ at seed dressing of level of 0 mL kg^−1^ showed 2.4, 1.2 and 16.6 % higher nodes per plant as compared to 0, 10 and 30 L ha^−1^, respectively, while at a seed dressing of 100 mL kg^−1^, the increase was 3.5, 3.5 and 16.0%, respectively (Figure 2). The maximum increase in potassium humate soil application at the rate of 20 L ha^−1^ was found at seed dressing of 200 mL kg^−1^ which was 9.8, 3.5 and 28.1% as compared to 0, 10 and 30 L ha^−1^. The soil application at the rate of 30 L ha^−1^ showed 13.8, 12.0, 7.7 and 15.3% lower plant height at the rate of seed dressing levels of 0, 100, 150 and 200 mL kg^−1^, respectively. The main interaction effects of potassium humate soil and seed dressing application was found to be significant at *p* < 0.05 (Table 2). The potassium humate soil application at the rate of 30 L ha^−1^ showed a lower sympodial branches per plant as compared to other levels (0, 10 and 20 L ha^−1^). However, soil potassium humate at the rate of 20 L ha^−1^ showed a maximum sympodial branches per plant as compared to 0, 10 and 30 L ha^−1^ (Figure 2). However, all potassium humate soil application levels showed better results at a seed dressing at the rate of 200 mL kg^−1^. The plant height, number of nodes and number of sympodial branches showed significant positive pearson correlation with each other (Figure 3).

### 2.4. No. of Bolls Per Plant, Boll Weight and Seed Cotton Yield

**Figure 4:**
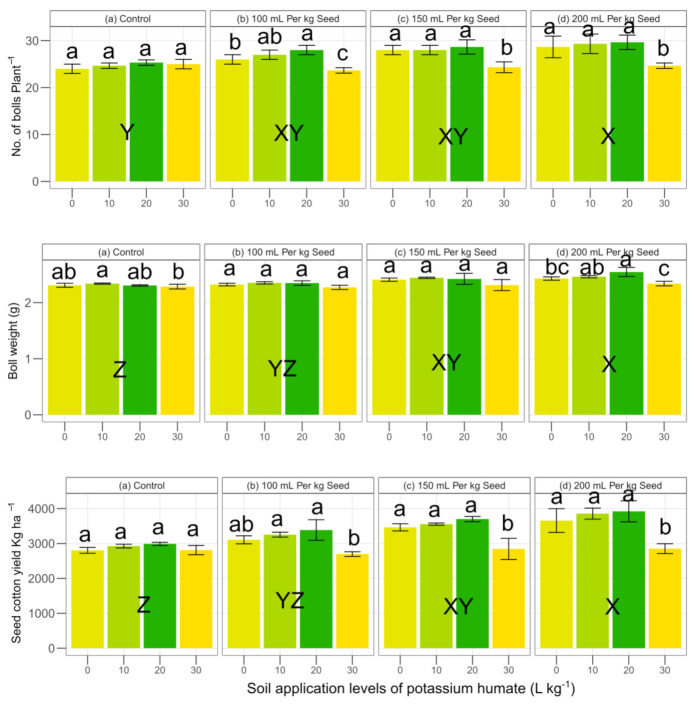
Effect of potassium humate on the number of bolls plant^−1^, average boll weight and the yield of seed cotton. Within the seed dressing application, the same lowercase letter (s) a, b, or c, indicates that the soil potassium humate application rates are not statistically different from each other at *p* < 0.05. Among the seed dressings, the same uppercase letter (s) W, X, Y or Z, showed a non-significant effect. Error bars represent the standard deviation of the mean (n = 3, three replications).

The main interactive effects of soil applied and seed dressed potassium humate were found to be significant at *p* < 0.05 on bolls per plant (Table 2). All levels of potassium humate (0, 10, 20 and 30 L ha^−1^) showed maximum results at a seed dressing of 200 mL kg^−1^. However, the performance of soil application of potassium humate at the rate of 20 L kg^−1^ showed higher numbers of bolls at all levels of seed dressing (0, 100, 150 and 200 mL kg^−1^) as compared to 0, 10 and 30 L ha^−1^ (Figure 4). However, the higher dose of soil application of potassium humate (30 L kg^−1^) showed a minimum response compared to the control at all levels of seed dressing. The main effect of potassium humate soil and seed dressing on the boll weight was found to be significant at *p* < 0.05. However, the interaction effect of potassium humate soil × potassium seed dressing was found to be non-significant at *p* < 0.05 (Table 2). The soil application of potassium humate at the rate of 10 L kg^−1^ performed better at seed dressing levels except 200 mL kg^−1,^ where soil potassium humate at the rate of 20 mL performed better. The soil potassium humate at the rate of 10 mL performed with a 1.3, 1.3, 1.2 and 1.2% increase over the control at seed dressing levels of 0, 100, 150 and 200 mL kg^−1^, respectively as compared to the control. However, all levels of soil potassium humate (0, 10, 20 and 30 L ha^−1^) performed better at a potassium humate seed dressing of 200 mL kg^−1^ (Figure 4). The main interaction effect of soil and seed dressing of potassium humate was found to be significant at *p* < 0.05 on the seed cotton yield (Table 2). The lowest yield (2697 kg ha^−1^) was found where potassium humate in the soil at a rate of 30 L ha^−1^ × seed dressing at the rate of 100 mL kg^−1^ were applied as compared to all other combinations. The potassium humate at the rate of 30 L ha^−1^ showed an overall lower response to the seed cotton yield as compared to 0, 10 and 20 L ha^−1^ at all seed dressing levels (0, 100, 150 and 200 mL kg^−1^). However, the response of all levels of potassium humate was found to be higher at 200 mL kg^−1^ seed dressing. The soil application of 20 L kg^−1^ at a seed dressing of 200 mL kg^−1^ showed 7.2, 1.7 and 37.48% higher seed cotton yield as compared to 0, 10 and 30 L ha ^−1^ soil application rates, respectively. The number of bolls per plant, boll weight and seed cotton yield showed a significant positive Pearson correlation with each other (Figure 3).

### 2.5. Ginning out Turn and Fiber Length

**Figure 5:**
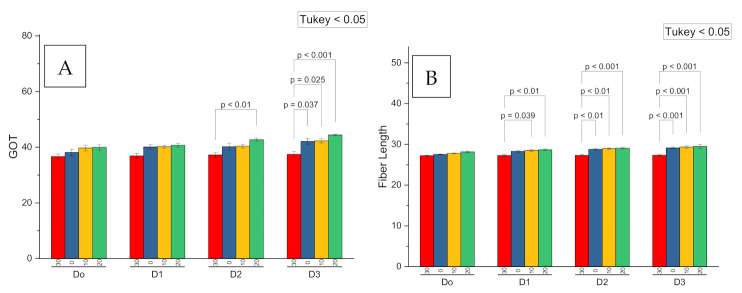
Probability values comparison for the seed dressed (D0 = 0, D1 = 100, D2 = 150 and D3 = 200 mL kg^−1^) and the soil applied potassium humate on the ginning out turn (**A**) and fiber length (**B**) of cotton.

The main interaction effects of the soil and seed dressing application of potassium on the ginning out turn (GOT) were found significant at *p* < 0.05 (Table 3; Figure 5A). The soil application of potassium humate at seed dressing levels of 200 mL kg^−1^ was found higher as compared to other levels of seed dressing (0, 100 and 150 mL kg^−1^). The soil potassium humate rate of 30 L kg^−1^ showed a lower GOT as compared to other levels (0, 10 and 20 L kg^−1^) at all seed dressing levels (Table 3; Figure 5A). The main interaction effect of soil and seed dressing of potassium humate was found significant at *p* < 0.05 on fiber length (Table 3; Figure 5B). All soil potassium humate rates showed a non-significant change in fiber length at the 0, 10 and 20 L kg^−1^ seed dressing level of potassium humate except 30 L kg^−1^ which was slightly lower at the 100, 150 and 200 mL kg^−1^ seed dressing level (Table 3). However, the higher response of all soil potassium humate rates was at 200 mL kg^−1^ seed dressing. Fiber length and GOT showed a significant positive Pearson correlation with each other (Figure 3).

### 2.6. Fiber Strength and Fitness

**Figure 6:**
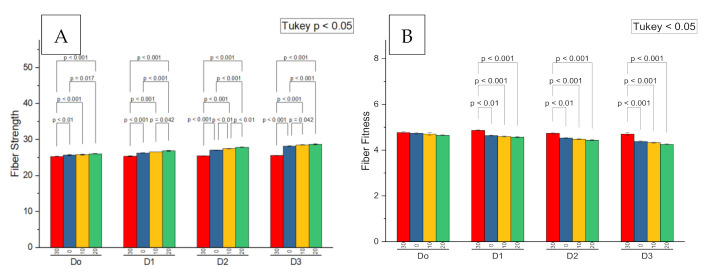
Probability values comparison for the seed dressed (D0 = 0, D1 = 100, D2 = 150 and D3 = 200 mL kg^−1^) and soil applied potassium humate on the fiber strength (**A**) and the fiber fitness (**B**) of cotton.

The main interaction effects of the soil and seed dressing potassium humate on the fiber strength were significant at *p* < 0.05 (Table 4; Figure 6A). The soil application at the rate of 20 L ha^−1^ showed a higher fiber strength on all the levels of seed dressing. The increase at mL kg^−1^ seed dressing was 1.4, 1.0 and 3.16, respectively, compared to 0, 10 and 30 L ha^−1^, respectively (Table 4). The increase at 100 mL kg^−1^ was 2.2, 1.3 and 5.9%, respectively, as compared to 10 and 30 L ha^−1^, respectively. The increase at 150 mL kg^−1^ was 5.4, 2.6 and 12.0% to as compared to 0, 10 and 30 L ha^−1^, respectively. The increase at 200 mL kg^−1^ was 1.8, 0.7 and 12% as compared to 0, 10 and 30 L ha^−1^ (Table 4). All levels of soil potassium humate performed better at a higher level of potassium humate seed dressing (200 L kg^−1^). The main interaction effect of soil and seed dressing potassium humate was found to be significant on the fiber fineness at *p* < 0.05 (Table 4; Figure 6B). The fiber fineness was found to be higher at 0 seed dressing as compared to 100, 150, and 200 mL kg^−1^ (Table 4). The maximum fiber fineness (4.86) was found where the soil potassium humate and seed dressing of 30 L ha^−1^ and 100 mL kg^−1^ were applied, respectively. The minimum fiber fineness (4.253) was found at 20 L ha^−1^ soil potassium humate and 200 mL kg^−1^ seed dressing (Table 4). Fiber strength and fiber fitness showed a significant negative Pearson correlation with each other (Figure 3).

## 3. Discussion

The current field study was conducted to evaluate the impact of different rates of soil and seed dressing of potassium humate on soil properties, cotton productivity and fiber quality attributes. The results of this study showed that the application of potassium humate improved the nutrient status of the soil. In addition, the results also revealed that the growth, yield and fiber quality were low where no seed dressing of potassium humate was applied. Furthermore, the data of the experiment showed that the soil application rate of potassium humate exceeding 20 L ha^−1^ resulted in low values of cotton productivity even compared to where no seed dressing and soil application of potassium humate was performed. The soil application of potassium humate was more effective at higher seed dressing levels of potassium humate (200 L kg^−1^) compared to lower rates 0, 100 and 150.

The application of potassium humate in the current study reduced the soil alkalinity and pH while it increased the organic matter, available phosphorus and extractable potassium, as compared to where no soil potassium humate was applied. The higher application rates showed more improvement in soil properties as compared to lower rates of potassium humate application. This could be due to the improvement of soil structure by the use of soil potassium humate as reported in a study of Imbufe et al. [35]. The use of potassium in the study of Lmbufe et al. [35] improved the soil aggregate stability by the formation of a clay–humate complex which protected the soil particles from disaggregation. The improvement in soil structure reduced the infiltration of soil water. The low infiltration of water could enhance the availability of nutrients by reducing their losses. In another study, Shujrah et al. [36] found that potassium humate at the rate of 100 kg ha^−1^ increased the soil exchange capacity (CEC) by 17.2% compared to the control. The increase in CEC may be a reason of increased nutrient contents in the current study. Furthermore, potassium humate is the stable humus and it provides exchange sites for nutrient absorptions [3]. Hence, the use of potassium humate reduces the leaching of nutrients by absorption on clay sites due to higher CEC and increases its availability to plant growth.

The increase in cotton growth, yield and fiber quality in the treatments where the seed dressing of potassium humate was applied as compared to where no seed dressing at present may be due to the increase in the germination percentage and protection from the seed borne diseases. In the study of Hassanpana and Khodadadi [37], 70% germination of potato seed was found with potassium humate seed treatment while the germination with water and control treatment was 31 and 15%, respectively. In another study conducted by Bostan et al. [38], we found higher germination (53.33%) for the 0.02 mL/seed treatment as compared to the seeds without seed dressing treatment. A poor rate of photosynthesis due to the deficiency of K [39,40] in cotton resulted in the synthesis of reactive oxygen species and nitrogen assimilation [41,42]. This also decreased the loading speed of phloem and carbohydrates starvation that played an imperative role in cotton bolls development. Difficulty in carbohydrate acquisition decreased the fiber strength and cotton lint yield [42,43,44].

The potassium humate application exceeding 20 kg ha^−1^ (30 kg L^−1^) showed a lower response as compared to 0, 10 and 20 L ha^−1^ on the cotton productivity in current study. This may be due to the excess absorption of nutrients on the surface of humus and their restricted availability to plants. The number of studies in the literature also confirmed that the low application rate of potassium humate performed better on the soil properties and crop productivity. In a study of Patil et al. [32], the application of 5 kg ha^−1^ potassium humate showed better results compared to non-application of potassium humate on soybean plant height, the number of nodules, number of pods and yield. In another study, Hemida et al. [45] found an improvement in the soil physiochemical characteristics and productivity of Phaseolus vulgaris with the soil application of 0.5 kg^−1^ potassium humate and 1.0 mM-α-tocopherol as compared to the control. Baraldi et al. [46] found that the higher application of potassium 500 mg kg^−1^ in an in vitro medium showed a lower root length and root system of golden delicious as compared to the lower rates of potassium humate application (0, 50 and 250 mg L^−1^). The strong and positive correlations were found among the growth, yield and fiber qualities except fiber length (Table 5).

The soil potassium humate application performed better where the seed dressing of 200 mL kg^−1^ was applied in the current study. This may be due to the better germination and protection from seed born disease due to the seed dressing of potassium humate and the better nutrient availability and uptake of nutrients through soil potassium humate application [37]. Therefore, the combined seed dressing and soil application of potassium humate provided better soil environments for higher cotton productivity as compared to the individual application of soil or seed dressing of potassium humate [45].

## 4. Materials and Methods

### 4.1. Site Description

A field experiment was conducted at the Agronomic Research Area, Muhammad Nawaz Sharif University of Agriculture and Multan, Pakistan (30.1536° and 71.4457°) during the cotton growing season in 2018. The land elevation from sea levels is about 122 m. The soil was loamy textured (33% sand, 37% silt and 30% clay) with EC*e* 3500 µS cm^−1^ [47] (Ohaus Starter ST3100C-B Bench Conductivity Meter), pH 8.5 [48] (Jenway 3510 pH Mete), organic matter content 0.56% [49] (potassium dichromate oxidation and titration with ferrous ammonium suphate), phosphorus 6.70 mg kg^−1^ [50] (Hitachi U-2000 UV Spectrophotometer), cation exchange capacity (CEC) 250 mmol(+) kg^−1^ [51] and potassium 210 mg kg^−1^ soil [52] (Jenway PFP7 Flame Photometer) (Table 5).

### 4.2. Treatments

The potassium humate (Porus, FMC Company; humic acid 10% + K_2_O 3.5% *w*/*v*) was applied as seed dressing and soil application. The seed dressing of potassium humate was done at the rate of 0, 100, 150 and 200 mL kg^−1^ seed and soil application at the rate of 0, 10, 20 and 30 L ha^−1^ (Table 5). For seed dressing, fuzzy cotton seed was placed overnight in plastic wash-tubs containing different concentrations of potassium humate solutions, while in the control treatment, the seed was subjected to distilled water treatment. Continuous air was circulated in the tubs to supply oxygen to the submerged seed. In the soil, the prescribed levels of potassium humate were applied with irrigation to 40 days old crop.

### 4.3. Field Experiment

After wheat harvesting, the field was prepared by ploughing the soil 2–3 times followed by planking. The seed beds were prepared by a bed shaper. Cotton seed (*Gossypium hirsutum* L.) variety MNH-1026 was planted manually by dibbler method on 24 May 2018. A row × row distance of 75 cm and plant × plant of 22 cm were maintained. The recommended nitrogen, phosphorus and potassium at the rate of 115–60–60 kg ha^−1^ as urea, diammonium phosphate (DAP) and potassium sulphate (K_2_SO_4_) were applied, respectively. The full dose of phosphorus and potassium was applied through broadcasting at the time of sowing, while nitrogen was applied in three equal splits at 40, 60 and 80 days old crop. A total of nine irrigations were applied throughout the cotton sowing season. The first irrigation was applied at planning while the following irrigations were applied according to the soil moisture contents. The pre-emergence weeds were controlled through weedicide (Dual Gold at the rate of 500 mL acre^−1^) and the post-harvest weeds were removed by manual hoeing. The pesticides (Flunicamid, Buprofezin,), Diafenthuron, Lamdacyhalothrin) were sprayed to maintain the pest infestation under the economic threshold level. The crop was harvested on 24^th^ November 2018 when 80% bolls were matured.

### 4.4. Data Collection

Plant height, nodes per plant, monopodial and sympodial branches were measured from five selected plants from each plot. The seed cottons were picked from the whole plot manually and then the yield was converted into Mg ha^−1^. The bolls were air dried to obtain the moisture contents below 11% and the weight of individual boll was measured. A representative tester of 100 g from all treatments plot was used for ginning. Ginning percentage (%) was found by using the following formula [53]:

$$\mathrm{GOT}\;\left(\%\right)=\;\frac{\mathrm{Weight}\;\mathrm{of}\;\mathrm{lint}\;\left(g\right)}{\mathrm{Weight}\;\mathrm{of}\;\mathrm{seed}\;\cot\mathrm{ton}}\times 100$$

The fiber length, fiber strength and fiber fineness were measured by placing a 2.0 g sample of lint in High Value instruments (HVI) available in Fibre Technology Section, Central Cotton Research Institute, Multan, Pakistan.

### 4.5. Statistical Analysis

Data on the growth, yield and fiber quality parameters were statistically analyzed using R software [54]. The seed dressing of potassium humate was considered as a fixed effect while the soil application rates of potassium humate were considered a random effect. The means were compared at *p* < 0.05, using the adjusted Tukey multiple comparison procedure with “emmeans” package.

## 5. Conclusions

The results of the present study revealed that the potassium humate soil application and seed dressing treatment improved the soil properties, cotton productivity and fiber quality. However, the soil application of the potassium performed best with the highest seed dressing of potassium humate (200 mL kg^−1^). The soil application of potassium humate at the rate of 20 L ha^−1^showed higher results at all levels of seed dressing (0, 100, 150 and 200 mL kg^−1^). However, the potassium application rate at the rate of 30 L ha^−1^ showed lower results as compared to 0, 10 and 20 L ha^−1^. Therefore, it is recommended that the soil application of potassium humate should not exceed 20 L ha^−1^ when the seed dressing of potassium humate is applied.

## Figures and Tables

**Table 1:** The post-experiment soil properties of the experimental site.

Soil Applied Potassium Humate	Electrical Conductivity	pH	Organic Matter	Nitrogen	Available Phosphorus	Exchangeable Potassium
L ha^−1^	µS cm^−1^		5	g kg^−1^	mg kg^−1^	mg kg^−1^
0	3020	8.40	0.62	0.31	7.06	195.00
10	3010	8.45	0.64	0.32	7.25	207.00
20	2890	8.45	0.66	0.33	7.52	212.5
30	2710	8.37	0.67	0.34	7.95	222.5

**Table 2:** The main interactive effects of soil and seed dressing potassium humate on cotton growth, yield and fiber quality attributes at *p* < 0.05.

Effect	Plant Height	Nodes Plant^−1^	Sympodial Branches	Bolls Plant^−1^	Boll Weight	Seed Cotton Yield	GOT	Fiber Length	Fiber Strength	Fiber Fitness
PH soil application (S)	0.001	0.001	0.001	0.001	0.001	0.001	0.001	0.001	0.001	0.001
PH seed dressing (D)	0.001	0.001	0.001	0.001	0.001	0.001	0.001	0.001	0.001	0.001
S × D	0.001	0.220	0.001	0.020	0.210	0.010	0.001	0.001	0.001	0.001

GOT: Ginning Out Turn.

**Table 3:** The effect of the soil applied and seed dressed potassium humate on the ginning out turn (GOT) and fiber length.

Soil Application (L ha^−1^)	GOT (%)				Fiber Length (mm)			
	Seed Dressing (mL kg^−1^)				Seed Dressing (mL kg^−1^)			
	0	100	150	200	0	100	150	200
0	38.1 ± 1.87 a	40.1 ± 1.47 a	40.2 ± 2.13 ab	42.1 ± 1.81 a	27.53 ± 0.15 a	28.30 ± 0.4 ab	28.8 ± 0.35 a	29.13 ± 0.35 a
10	39.73 ± 1.72 a	40.2 ± 0.87 a	40.4 ± 1.11 ab	42.3 ± 1.35 a	27.8 ± 0.10 a	28.50 ± 0.4 a	28.97 ± 0.31 a	29.33 ± 0.55 a
20	39.97 ± 1.65 a	40.7 ± 1.22 a	42.73 ± 10 a	44.4 ± 0.46 a	28.17 ± 0.31 a	28.7 ± 0.36 a	29.07 ± 0.40 a	29.5 ± 0.79 a
30	36.67 ± 1.55 a	36.9 ± 1.51 a	37.23 ± 1.47 b	37.43 ± 1.72 b	27.23 ± 0.15 a	27.27 ± 0.45 b	27.3 ± 0.36 b	27.33 ± 0.38 b

The values are the mean and standard deviations of three replications. Within each seed dressing, the level values with similar letter (s) are not statistically different from each other at *p* < 0.05.

**Table 4:** The effect of the soil applied and the seed dressed potassium humate on fiber strength and fiber fitness.

Soil Application (L ha^−1^)	Fiber Strength (g/tex)				Fiber Fineness (mic)			
	Seed Dressing (mL kg^−1^)				Seed Dressing (mL kg^−1^)			
	0	100	150	200	0	100	150	200
0	25.73 ± 0.15 b	26.30 ± 0.10 b	27.10 ± 0.10 c	28.20 ± 0.10 b	4.73 ± 0.06 a	4.63 ± 0.04 b	4.53 ± 0.04 b	4.38 ± 0.05 b
10	25.83 ± 0.15 ab	26.57 ± 0.06 b	27.50 ± 0.10 b	28.53 ± 0.06 a	4.70 ± 0.10 a	4.59 ± 0.04 b	4.48 ± 0.04 b	4.33 ± 0.04 b
20	26.10 ± 0.10 a	26.90 ± 0.10 a	27.90 ± 0.10 a	28.73 ± 0.15 a	4.65 ± 0.05 a	4.57 ± 0.04 b	4.43 ± 0.04 b	4.25 ± 0.04 b
30	25.30 ± 0.10 c	25.40 ± 0.10 c	25.50 ± 0.10 d	25.60 ± 0.10 c	4.77 ± 0.06 a	4.86 ± 0.04 a	4.73 ± 0.06 a	4.70 ± 0.10 a

Values are the mean and standard deviations of three replications. Within each seed dressing level, values with similar letter (s) are not statistically different from each other at *p* < 0.05.

**Table 5:** The pre-experiment soil properties of the experimental site.

Soil Depth	pH	Electric Conductivity	Organic Matter	Available Phosphorus	Nitrogen	Exchangeable Potassium	CEC	Texture
cm	-	µS cm^−1^	%	mg kg^−1^	g kg^−1^	mg kg^−1^	mmol (+) kg^−1^	-
0–15	8.50	3500	0.56	6.70	0.28	210.00	250	Loam
15–30	8.40	2930	0.58	5.70	0.29	180.00	-	-

CEC: Cation Exchange Capacity.
